# Maternal detection of neonatal jaundice during birth hospitalization using a novel two-color icterometer

**DOI:** 10.1371/journal.pone.0183882

**Published:** 2017-08-24

**Authors:** Bolajoko O. Olusanya, Tina M. Slusher, Donald O. Imosemi, Abieyuwa A. Emokpae

**Affiliations:** 1 Center for Healthy Start Initiative, Lagos, Nigeria; 2 Department of Pediatrics, Division of Global Health, University of Minnesota, Minneapolis, Minnesota, United States of America; 3 Hennepin County Medical Center, Minneapolis, Minnesota, United States of America; 4 Lagos Island Maternity Hospital, Lagos, Nigeria; 5 Massey Street Children’s Hospital, Lagos, Nigeria; University of Oklahoma, UNITED STATES

## Abstract

**Background:**

Mothers are frequently the first to observe the onset of jaundice in their newborn infants before the decision to seek treatment. However, simple-to-use tools that could facilitate early detection of jaundice and assist mothers to seek professional care, especially after hospital discharge, are rare. This study therefore, set out to evaluate the performance of a -two-color icterometer (Bilistrip™) as a possible screening tool for detecting significant jaundice by mothers or care-givers in the first week of life.

**Methods:**

Prior to discharge, mothers in a maternity hospital were trained to use the Bilistrip™ on the blanched skin of their baby’s nose to ascertain absence (Light Yellow) or presence (Dark Yellow) of significant jaundice. Their babies had transcutaneous bilirubin (TcB) measurements independently, along with total serum bilirubin (TSB) if indicated. The reliability of Bilistrip™ as a screening test for significant jaundice was determined at different TcB and TSB thresholds. The predictive performance of Bilistrip™ was also evaluated with multivariable logistic regression.

**Results:**

Some 2492 mother-infant pairs were enrolled over 15 months, of which 347 (13.9%) chose Dark Yellow. The mean TcB for Dark Yellow (10mg/dL) was significantly higher (p<0.001) than for Light Yellow (6.1mg/dL). Bilistrip™ showed increasing sensitivity (47.0% - 92.6%) and negative predictive value (NPV) (91.4% - 99.9%) for selected TcB thresholds (≥10mg/dL, ≥12mg/dL, ≥15mg/dL, and ≥17mg/dL). Among neonates with TSB measurements (n = 124), Bilistrip™ was associated also with increasing sensitivity (86.8% - 100%) and NPV (62.5% - 100%). The sensitivity and NPV for detecting neonates requiring phototherapy were 95.8% respectively. Only one of the 24 neonates who required phototherapy was missed by the Bilistrip™.

**Conclusions:**

Bilistrip™ is a potential decision-making tool for empowering mothers to detect neonates with clinically significant jaundice that may require close monitoring or treatment, and neonates not requiring treatment for jaundice in the first week of life.

## Introduction

Hyperbilirubinemia, manifesting as jaundice, affects about 60–80% of all newborns and is a leading cause of hospital admission or re-hospitalization in the first week of life [1–3]. Mothers are frequently the first to observe jaundice in the vast majority of the affected newborns, especially outside hospital settings [4,5]. Maternal role is even of more interest in low-and middle-income countries with a high prevalence of risk factors for neurotoxicity and a high proportion of deliveries outside hospitals [6]. However, poor recognition of jaundice and delayed presentation to hospital remain major contributors to the burden of bilirubin encephalopathy, excessive rates of avoidable exchange transfusion and related adverse outcomes [1,7]. Maternal education and empowerment for timely detection of jaundice is therefore, crucial for effective community-based interventions needed to curtail the burden of severe neonatal jaundice [8–10].

Few studies have investigated maternal use of visual aids to detect jaundice in neonates [11–13]. Perhaps, the most widely reported tool is the Perspex icterometer (transcutaneous jaundice meter) pioneered by Dr. Gosset in 1960 primarily for use by health workers in hospital settings [14]. This device consists of a plastic strip with transverse yellow stripes of increasing intensity to reflect different severity of jaundice, on a scale from 1 to 5 when placed on the blanched skin of an infant’s nose. A variant of this strip, with eight transverse yellow stripes has been reported recently as a potential screening tool for mothers [13]. However, differentiating and matching the skin color of a newborn with several close shades of yellow may be difficult for untrained observers including mothers and care-givers under variable lighting conditions. This study, therefore set out to evaluate the predictive performance of a two-color transcutaneous icterometer (Bilistrip™) as a possible dichotomous (“pass” or “refer”) screening tool for identifying neonates with clinically significant jaundice in home settings.

## Methods

**Fig 1:**
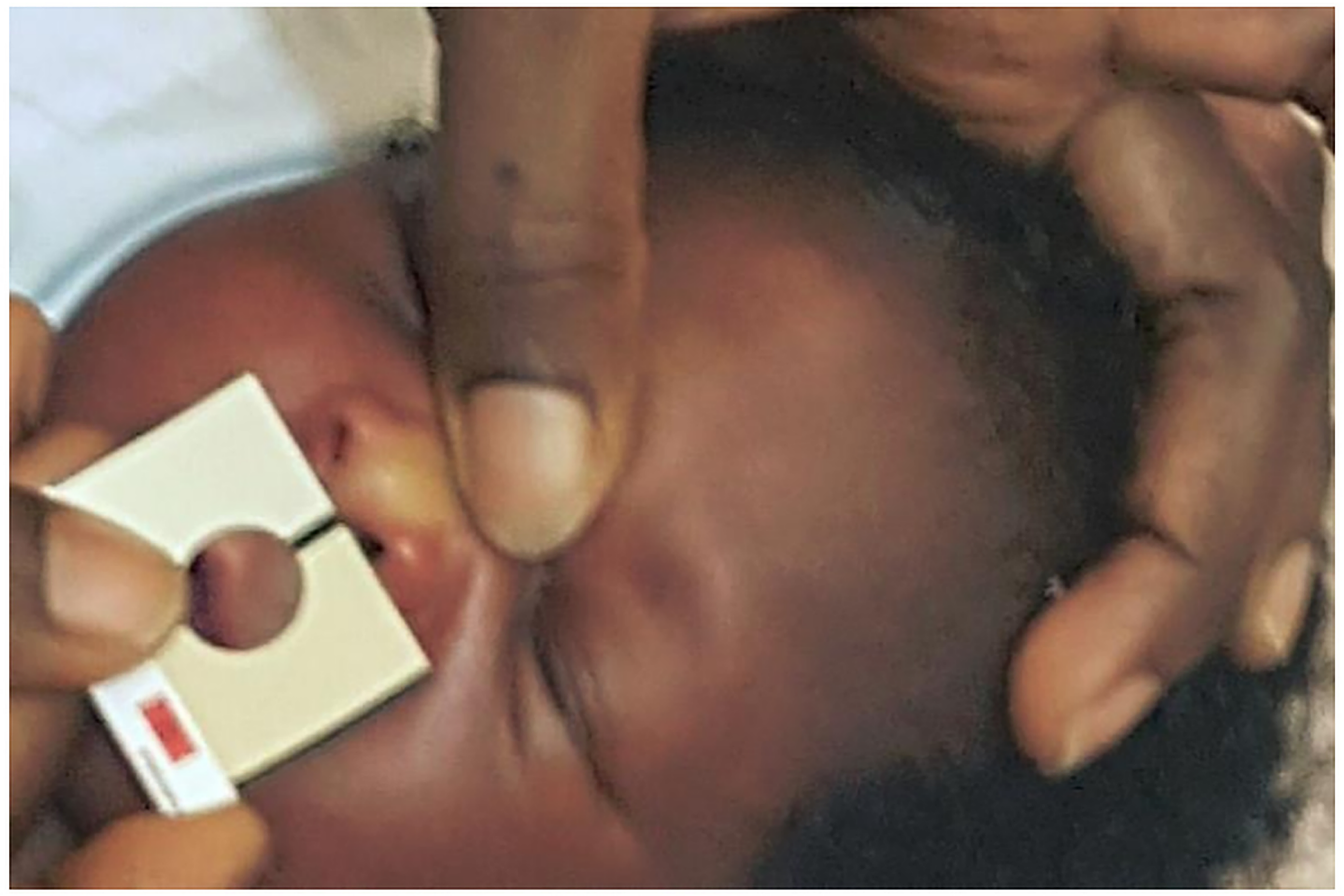
Baby tested with Bilistrip™ by a mother.

This prospective study was guided by the Standards for Reporting of Diagnostic Accuracy Studies (STARD) statement [15], and conducted at Island Maternity Hospital (IMH) in Lagos, Southwest, Nigeria. Eligible study participants were mothers of late-preterm and term neonates (gestational age ≥35weeks or birthweight >2.2kg) in the well-baby nursery. They were consecutively enrolled between August 2015 and October 2016. Newborns with congenital disorders, skin abnormalities, treated by phototherapy, with sick or non-consenting mothers and multiple births were excluded. Prior to enrolment, a dedicated research staff explained the importance of the study in assisting the mother to detect possible significant jaundice in the newborn for prompt intervention using Bilistrip™. This index test was developed with two shades of yellow by Bilimetrix-USA (23304 Locust Way, Bothell WA, USA), and modelled after Ingram Icterometer (Fig 1). The dimensions are 3.5 x 3.0 cm, with 1.3 cm diameter hole.

Consenting mothers received a brief demonstration on how to blanch the skin on the bridge of their baby’s nose with digital pressure to reveal underlying color matched to two options (Light Yellow or Dark Yellow) on the Bilistrip™, under sufficient ambient lighting condition. The Light Yellow (color A) was intended to depict absence of significant jaundice, while the Dark Yellow (color B) depicted presence of significant jaundice. The mother was then requested to record the color that matched the baby’s blanched skin better on a separate record card designed for the study. A participating mother did not have to make a choice if she was undecided or unsure. Thereafter, the baby’s transcutaneous bilirubin (TcB) level was determined by another research staff (blinded to Bilistrip™ results), using JM-103^®^ bilirubinometer (Draeger Medical Telford, PA), in accordance with the manufacturer’s instructions. As per the previously reported study protocol [16], total serum bilirubin (TSB) measurement was required if TcB level reached 3mg/dL below age-specific threshold for phototherapy recommended by the American Academy of Pediatrics (AAP) [17]. Approximately one hour, after the TcB measurement, TSB was determined from heparinized capillary blood samples analyzed by direct spectrophotometry using the Advanced Bilirubin Stat-Analyzer Model-BR2 (Advanced Instruments Inc, Norwood, MA). Only measurements obtained prior to phototherapy were considered for this study.

### Test performance criteria

Our primary objective was to evaluate the ability of Bilistrip™ to detect neonates with TcB thresholds of ≥10 mg/dL (171 μmol/L), ≥12 mg/dL (205 μmol/L), ≥15 mg/dL (256 μmol/L), and ≥17 mg/dL (291 μmol/L). Additionally, we sought to determine the predictive ability of Bilistrip™ to identify the risk of jaundice at each of the specified thresholds after adjusting for potential confounders. Demographic data for mother-infant pairs and other putative confounding factors were elicited from mothers or retrieved from hospital records. These included maternal age, ethnicity, parity, mode of delivery, infant’s gender, birthweight, gestational age and age on enrollment. Skin color was not considered as it had been shown to lack association with TcB in this study population, as previously reported [18]. Significant jaundice or hyperbilirubinemia was defined as TcB or TSB ≥12 mg/dL, typically within the first 48 hours of life, consistent with the local practice that reflected the high prevalence of glucose 6-phospho-dehydrogenase (G6PD) deficiency in the population [19].

Using a 7% prevalence of severe jaundice in this population [19], and an expected sensitivity of 90% for Bilistrip™, we estimated that a minimum sample size of 1976 mother-infant pairs would be required, within 5% margins of error, at 95% CI [20]. This would also allow 90% power for the target sensitivity.

### Ethics statement

This study was conducted under the institutional ethical approval (Reference No: SHMB/728/Vol. VI, dated 23 January 2015) from Lagos State Health Service Commission, the Ethics Review Board of all hospitals owned and managed by the Lagos State Government of Nigeria. Informed consent from the parents were obtained in writing prior to enrollment of the newborn using a duly approved consent form. All patient records were anonymized and de-identified prior to analysis. The individual in this manuscript (Fig 1) has given written informed consent (as outlined in PLOS consent form) to publish these case details.

### Statistical analysis

We used both TcB and TSB as reference measures. The use of TcB was intended to guide the care-givers in settings where timely and accurate TSB determination cannot be readily assured. We computed the sensitivity, specificity, positive predictive value (PPV), negative predictive value (NPV), positive likelihood ratio (PLR) and negative likelihood ratio (NLR) along with corresponding 95% confidence interval (CI) for the specific thresholds of interest. Since only one pair of measurements for each participant was considered, we evaluated the predictive ability of Bilistrip™ to identify the risk of jaundice at each of the specified thresholds using multivariable logistic regressions. All the elicited covariates were included in the regression analyses based on biological plausibility. The estimated adjusted odds ratio (OR), was regarded as an approximation of the relative risk. The discriminatory performance of each threshold-specific logistic model was assessed with c-statistic [21]. The calibration of each adjusted model was also assessed with Hosmer-Lemeshow goodness-of-fit test. Lastly, the TSB performance of Bilistrip™, including AAP criteria for phototherapy, was explored among neonates with paired TcB-TSB measurements. All tests of significance were two-tailed at alpha level of p<0.05. IBM SPSS software, Version 23.0 (IBM Corporation, Armonk, NY) was used for all statistical analyses.

## Results

A total of 2528 mother-infant pairs were enrolled over the study period, of which 36 (1.4%) mothers who were undecided were excluded from full analysis (S1 Fig). Of the remaining 2492 participants, 2145 (86.1%) chose color A and 347 (13.9%) color B. The characteristics of participants are summarized in Table 1. Over half of the mothers were 20–35 years of age, of Yoruba ethnicity, multiparous and delivered vaginally. More than half of the infants were male, weighed between 2.5–3.0 kg at birth, and were full-term. The majority (78.6%) were enrolled by 48 hrs. after birth, and only 7 mothers were recruited after day 7.

**Table 1:** Characteristics of mother-infant pairs enrolled.

Factors	Total	Color B
	n = 2492 (%)	n = 347 (%)
**Maternal age (years)**		
<20	45 (1.8)	9 (20.0)
20–35	2090 (83.9)	289 (13.8)
>35	357 (14.3)	49 (13.7)
Mean ± SD [Range]	29.9 **±** 5.2	[15–55]
**Ethnicity**		
Hausa	136 (5.5)	24 (17.6)
Igbo	373 (15.0)	58 (15.5)
Yoruba	1663 (66.7)	212 (12.7)
Others	320 (12.8)	53 (16.6)
**Parity**		
Primiparous	1053 (42.3)	176 (16.7)
Multiparous	1439 (57.7)	171 (11.9)
**Mode of delivery**		
Vaginal	1377 (55.3)	212 (15.4)
Cesarean	1115 (44.7)	135 (12.1)
**Gender**		
Female	1148 (46.1)	159 (13.9)
Male	1344 (53.9)	188 (14.0)
**Birthweight (kg)**		
<2.5	192 (7.7)	22 (11.5)
2.5–3.0	852 (34.2)	108 (12.7)
>3.0	1448 (58.1)	217 (15.0)
Mean ± SD	3.2 **±** 0.5	3.2 **±** 0.5
**Gestational age (weeks)**		
<38	408 (16.4)	46 (11.3)
≥38	2084 (83.6)	301 (14.4)
Mean ± SD	38.5 **±** 1.6	38.5 **±** 1.6
**Postnatal age (days)**		
0–2	1959 (78.6)	258 (13.2)
3–7	526 (21.1)	88 (16.7)
>7	7 (0.3)	1 (14.3)
Mean ± SD	1.9 **±** 1.2	2.0 **±** 1.2

**Fig 2:**
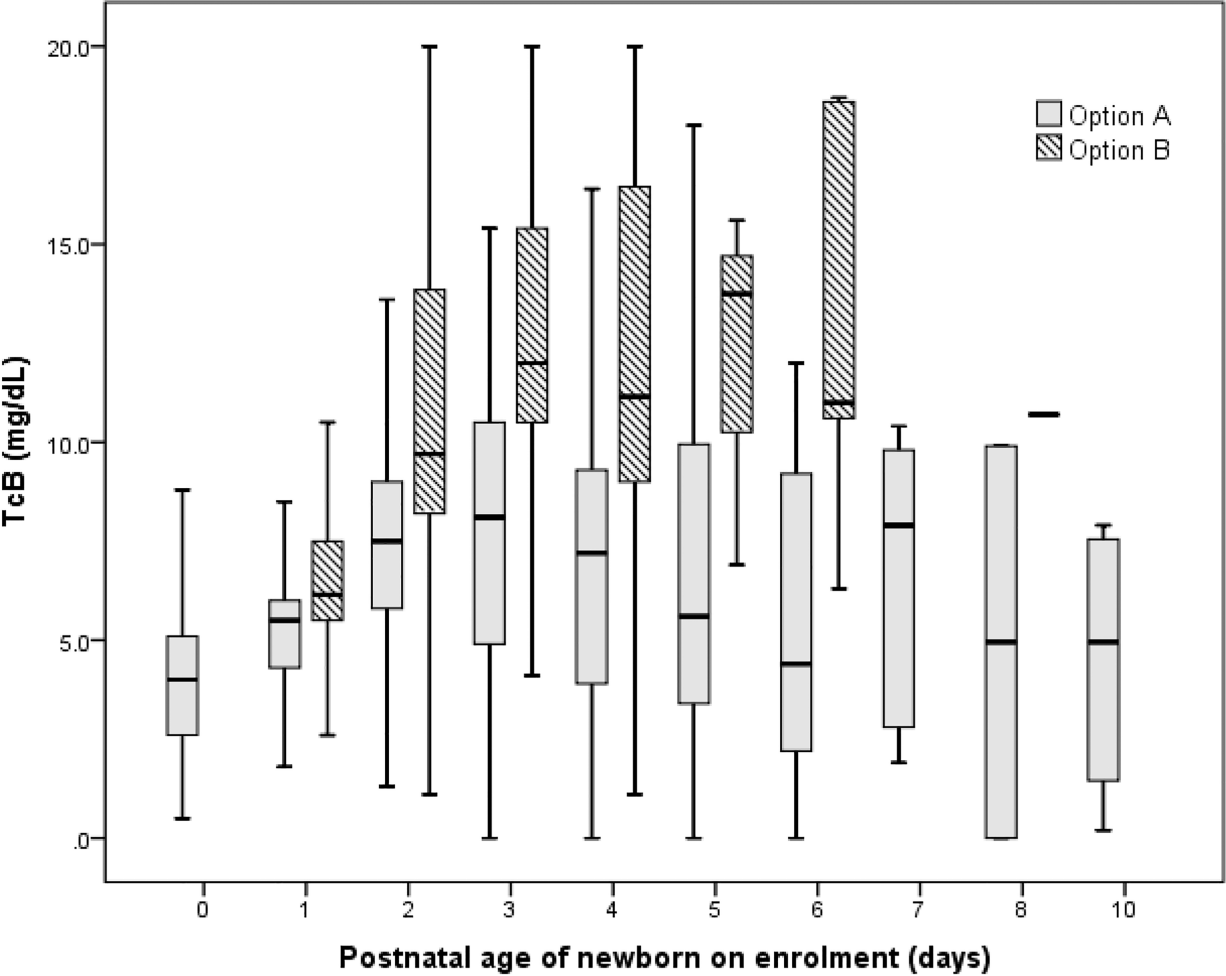
Age-specific TcB levels for the two-color options on Bilistrip™.

Among all neonates, TcB was ≥10 mg/dL in 347 (13.9%), ≥12 mg/dL in 167 (6.7%), ≥15 mg/dL in 65 (2.6%), and ≥17 mg/dL in 27 (1.1%). Overall mean TcB for color B (10.1 ± 4.2 mg/dL) was significantly higher than for color A (6.1 ± 2.7 mg/dL), p<0.001. Age-specific pattern also showed a consistently higher TcB level for color B, than color A, even for the only one infant with significant jaundice (color B) in the second week of life (Fig 2). TSB was determined in 124 (5.0%) neonates, with a mean of 11.1 ± 3.8 mg/dL for color B, compared with 9.1 ± 2.7 mg/dL for color A. In this group, TSB was ≥10 mg/dL in 68 (54.8%), ≥12 mg/dL in 35 (28.2%), ≥15 mg/dL in 15 (12.1%), and ≥17 mg/dL in 7 (5.6%). A total of 24 (19.4%) neonates met AAP criteria for phototherapy and 3 (2.4%) required exchange transfusion. The 36 excluded neonates had mean TcB of 7.0 ± 3.4 mg/dL. TSB was indicated in 3 neonates (range: 7.7–11.8 mg/dL), none of whom required phototherapy.

The reliability of Bilistrip™ compared to TcB is presented in Table 2. The sensitivity (47.0% - 92.6%), NPV (91.4% - 99.9%) and PLR (5.5–7.1) rose with increasing TcB threshold, with the best combination of test accuracy occurring at TcB ≥17 mg/dL. The NPV was quite high for all thresholds. Neonates of mothers who chose color B had substantial risk of having hyperbilirubinemia with or without adjustment for confounding variables (Table 3). The discriminatory ability for Bilistrip™ was highest for TcB ≥17 mg/dL (c-statistic = 0.94, 95% CI: 0.90–0.99). Models for predicting all TcB thresholds were well calibrated (Hosmer-Lemeshow test: p>0.05).

**Table 2:** Predictive performance of Bilistrip™ for different TcB thresholds (n = 2492).

Outcome	N	Sensitivity	Specificity	PPV	NPV	PLR	NLR
		(95% CI)	(95% CI)	(95% CI)	(95% CI)	(95% CI)	(95% CI)
**≥10mg/dL**	347	47.0	91.4	47.0	91.4	5.5	0.6
		(41.7–52.2)	(90.2–92.6)	(41.7–52.2)	(90.2–92.6)	(4.6–6.5)	(0.5–0.7)
**≥12mg/dL**	167	64.7	89.7	31.1	97.2	6.3	0.4
		(57.4–71.9)	(88.5–91.0)	(26.3–36.0)	(96.6–97.9)	(5.3–7.4)	(0.3–0.5)
**≥15mg/dL**	65	84.6	88.0	15.9	99.5	7.0	0.2
		(75.8–93.4)	(86.7–89.2)	(12.0–19.7)	(99.2–99.8)	(6.1–8.2)	(0.1–0.3)
**≥17mg/dL**	27	92.6	86.9	7.2	99.9	7.1	0.1
		(82.7–100)	(85.6–88.3)	(4.5–9.9)	(99.8–100)	(6.1–8.2)	(0.0–0.3)

**Table 3:** Multivariate association of Bilistrip™ with various TcB thresholds (n = 2492).

Outcome	Unadjusted OR	c-statistic	Adjusted OR	Hosmer-Lemeshow test	c-statistic
	(95% CI)	(95% CI)	(95% CI)	(95% CI)	(95% CI)
**≥10mg/dL**	9.4 (7.3–12.2)	0.69 (0.66–0.73)	13.4 (9.9–18.3)	p = 0.726	0.84 (0.81–0.86)
**≥12mg/dL**	16.0 (11.3–22.5)	0.77 (0.73–0.82)	19.4 (13.3–28.4)	p = 0.326	0.87 (0.84–0.90)
**≥15mg/dL**	40.2 (20.3–79.8)	0.86 (0.81–0.91)	43.0 (21.3–86.5)	p = 0.984	0.91 (0.87–0.95)
**≥17mg/dL**	83.2 (19.6–352.9)	0.90 (0.84–0.96)	91.2 (21.2–393.4)	p = 0.345	0.94 (0.90–0.99)

The performance of Bilistrip™ in identifying significant TSB thresholds is summarized in Table 4. The sensitivity (86.8% to 100%) and NPV (62.5% to 100%) improved with increasing TSB threshold, with the best combination of test accuracy occurring at TSB ≥17 mg/dL. The Bilistrip™ was associated with higher sensitivity in predicting TSB than TcB at all thresholds as shown in Table 2 and Table 4. The instrument also had a high sensitivity (95.8%) and NPV (95.8%) for neonates requiring phototherapy. Only one infant of the 24 that met the criteria for phototherapy was missed by a mother. Correlation between TcB and TSB was linear and statistically significant (Pearson’s r = 0.81, R^2^ = 0.65, p<0.001). However, the Bland-Altman plot showed a mean bias of 4.0 ± 2.2 mg/dL (95% limit of agreement: -0.3 to 8.3 mg/dL) between TcB and TSB (data not shown). Bilistrip™ was not associated with any adverse events, no subgroup analysis of variability was done, and there was no missing data.

**Table 4:** Predictive performance of Bilistrip™ for different TSB thresholds (n = 124).

Outcome	N	Sensitivity	Specificity	PPV	NPV	PLR	NLR
		(95% CI)	(95% CI)	(95% CI)	(95% CI)	(95% CI)	(95% CI)
**≥10mg/dL**	68	86.8	26.8	59.0	62.5	1.2	0.5
		(78.7–94.8)	(15.2–38.3)	(49.4–68.6)	(43.1–81.9)	(1.0–1.4)	(0.2–1.0)
**≥12mg/dL**	35	91.4	23.6	32.0	87.5	1.2	0.4
		(82.2–100)	(14.8–32.4)	(22.9–41.1)	(74.2–100)	(1.0–1.4)	(0.1–1.1)
**≥15mg/dL**	15	93.3	21.1	14.0	95.8	1.2	0.3
		(80.7–100)	(13.4–28.8)	(7.2–20.8)	(87.8–100)	(1.0–1.4)	(0.1–2.2)
**≥17mg/dL**	7	100	20.5	7.0	100	1.3	-
			(13.2–27.8)	(2.0–12.0)		(1.1–1.4)	
**AAP.PT**	24	95.8	23.0	23.0	95.8	1.2	0.2
		(87.8–100)	(14.7–31.2)	(14.7–31.2)	(87.8–100)	(1.1–1.4)	(0.1–1.3)

## Discussion

This study set out to evaluate the utility of a novel two-color icterometer, as a potential screening tool to aid mothers in detecting the onset of significant jaundice in their newborns. As a clinical decision-making tool, we considered it necessary to establish its usefulness in a hospital setting prior to discharge as a first step towards its experimentation and application in a home setting.

The overarching finding from this study was that visual estimation of jaundice with icterometer still has utility for detecting neonates with or at risk of hyperbilirubinemia, especially in settings where objective electronic devices for bilirubin estimation are not available routinely. Neonates suspected to have significant jaundice by their mothers (color B) were likely to have an elevated risk of hyperbilirubinemia and should be prioritized for further bilirubin assessment and monitoring for possible treatment. Neonates whose mothers selected color A may safely be regarded as not having clinically significant jaundice, except perhaps if hemolysis is suspected, which was not specifically investigated in this study. Another notable finding was the excellent sensitivity for identifying neonates who met AAP criteria for phototherapy.

Several studies have investigated maternal ability to recognize the presence of jaundice from the yellowish discoloration of baby’s skin and sclera [22–27]. However, the timing and accuracy of such recognition was highly variable. Maternal concern, often by those without any knowledge of jaundice, was more frequently prompted by early signs of acute bilirubin encephalopathy (ABE) such as feeding difficulty, irritability and restlessness of the infant [7]. Thus, our study would suggest that maternal use of a simple, user-friendly clinical aid like the Bilistrip™ may facilitate a faster and more proactive detection of jaundiced neonates who otherwise would have been detected late with established ABE. It is unlikely that mothers will be offered use of the more reliable but substantially more expensive electronic TcB devices for home-use even in high-income countries. For example, a typical TcB device costs about $3,000 per unit, excluding operating expenses, compared to $17 for the Ingram icterometer [11,28], or about $0.10 for a unit of Bilistrip™.

A few studies have evaluated mothers’ ability to detect the presence and severity of jaundice based on the extent of the cephalocaudal progression using Kramer chart and Ingram icterometer [11,29]. The two methods were found to have satisfactory correlation with actual TSB levels. However, both methods required respondents to select one out of five options. In one of the studies, among predominantly white neonates post-discharge, a cut-off of ≥2.5 on icterometer had a sensitivity of 73%, specificity of 65%, PPV of 44%, and NPV of 87% for identifying neonates with TSB ≥12 mg/dL; and sensitivity of 100%, specificity of 58%, PPV of 12%, and NPV of 100% for TSB ≥17 mg/dL [28]. In comparison, Bilistrip™ was associated with a sensitivity of 91%, specificity of 24%, PPV of 32%, and NPV of 88% for predicting TSB ≥12mg/dL; and sensitivity of 100%, specificity of 21%, PPV of 7%, and NPV of 100% for TSB ≥17mg/dL in our population. It would thus appear that race is not a confounding factor in the use of icterometers in newborns in the first week of life, as corroborated by other studies [28–32].

Several studies used ≥3 cut-off on Ingram icterometers for identifying neonates with significant jaundice [12,28,30,32]. The corresponding mean TSB for this cut-off value in most studies was approximately 10 mg/dL, which is comparable to the mean TcB of 10 mg/dL and mean TSB of 11mg/dL for color B. This finding thus supports the view that existing multi-shade icterometers can be substituted reliably by a simpler two-color icterometer like the Bilistrip™. In some studies, specific bilirubin levels were assigned to different shades of yellow on the icterometer [13]. But this practice may be inappropriate solely based on the findings in a single study [33].

Given the prospects of Bilistrip™ demonstrated in our study, the next challenge is how to optimize its usage by mothers at home (regardless of delivery place) to facilitate early presentation of neonates with or at risk of hyperbilirubinemia. Improved maternal knowledge does not always translate into improved care-seeking practices for neonatal jaundice. Nonetheless, several studies have demonstrated that maternal education has favorable impact on maternal recognition and care-seeking practices for neonatal jaundice [12,25,29,34]. Better understanding and empowerment will likely prevent most mothers from overlooking the potential risks of untreated or lately treated significant jaundice such as mortality and life-long disability in otherwise healthy newborns.

Notwithstanding, the reservations on visual estimation of jaundice especially among health workers [1], our study supports the view that icterometers hold promise as a low-cost intervention for curtailing the burden of neonatal jaundice in resource-poor countries [10]. Bilistrip™ lowers the cost of care even further to make it attractive for global child health consideration. While we do not expect mothers to take the lead in detecting jaundice during their hospital stay, in busy maternity units with limited nursing staff, maternal suspicion of significant jaundice may provide a valuable hint for health workers. It is important to emphasize that we do not expect that clinical decision for treatment will be based on the outcome of Bilistrip, particularly considering the small number of infants requiring phototherapy in this study. Rather that infants of mothers identified by Bilistrip as possibly having significant jaundice will undergo appropriate clinical assessment and further screening with non-invasive TcB before any blood draws for TSB.

A few limitations of this study deserve mention. Firstly, it was hospital-based, which may limit its generalizability at the population level. Secondly, the Bilistrip™ was specifically designed for use on the infant’s nose, as reported for Ingram icterometers in other studies [11,12,14,28,29,32]. We were therefore, unable to compare outcomes from different test sites on the infant’s body such as forehead, cheek, gum or sternum. Thirdly, because the study was conducted pre-discharge, predominantly within the first 2 days of life, the bilirubin levels among the participants were generally low and may not adequately reflect corresponding TcB or TSB values for the two options post-discharge. Fourthly, as with all icterometers, Bilistrip™ was unlikely to accurately capture rapidly rising TSB, as the rate of cutaneous bilirubin deposition does not always match the rate of rise in TSB. Lastly, it was unclear how the exclusion of serial daily recordings from the same mother would have impacted on findings in this study. Notwithstanding, Bilistrip™ offers a simpler alternative to the existing multi-color icterometers as a possible decision aid to facilitate timely recognition of jaundice by mothers before the onset of bilirubin encephalopathy.

## Conclusions

Our study suggests that mothers can be reliably empowered to identify the risk of hyperbilirubinemia with a simple, low-cost mechanical two-color icterometer like Bilistrip™ as an alternative to the current multi-yellow shaded icterometers. Infants detected by Bilistrip™ with suspected jaundice should be prioritized for further assessment by TcB and clinical examination before treatment. Additional studies are warranted to establish the likely impact of this decision aid for appropriate care-seeking practices for jaundiced neonates in home settings, particularly among mothers who delivered outside hospitals.

## Supporting information

S1 Fig Flow chart of study participants. (DOCX)

S1 Table STARD checklist for reporting of studies of diagnostic accuracy. (DOCX)

S1 Dataset (XLSX)

## Abbreviations

AAP: American Academy of Pediatrics
CI: Confidence interval
G6PD: Glucose 6-phospho-dehydrogenase
PPV: Positive predictive value
NPV: Negative predictive value
TcB: transcutaneous bilirubin
PLR: positive likelihood ratio
NLR: negative likelihood ratio
TSB: Total serum/plasma bilirubin
TCI: Two-color icterometer
