# Supplementary material for: Maternal detection of neonatal jaundice during birth hospitalization using a novel two-color icterometer
Source: PLoS One. 2017 Aug 24;12(8):e0183882. doi: 10.1371/journal.pone.0183882 (PMC5570328; doi:10.1371/journal.pone.0183882)
Supplement: S1 Fig — (DOCX) [file pone.0183882.s001.docx]

TcB: Transcutaneous Bilirubin, TSB: Total Serum Bilirubin

**S1 Fig. Flow chart of study participants**
